# Investigación científica prioritaria en Latinoamérica para orientar la prevención y el control de la COVID-19

**Published:** 2020-11-11

**Authors:** Zulma M. Cucunubá

**Affiliations:** 1 MRC Centre for Global Infectious Disease Analysis, Imperial College London, London, United Kingdom Departamento de Epidemiología Clínica, Pontificia Universidad Javeriana, Bogotá, D.C., Colombia zulma.cucunuba@imperial.ac.uk Pontificia Universidad Javeriana Pontificia Universidad Javeriana BogotáD.C. Colombia zulma.cucunuba@imperial.ac.uk

Pasados siete meses del primer caso (de infección por SARS-Cov-2 reportado en Latinoamérica y el Caribe, la región ha pasado a ocupar el primer lugar de muertes per cápita asociadas con la (COVID-19: para el 10 de octubre se habían reportado más de 350.000, lo (que representa el 34 % del total de las registradas globalmente ^1^. Además, el efecto de la pandemia en las economías de la región es el mayor de la historia en el último siglo ^2^, y varios de los países registran las tasas más altas de contracción económica ^3^. Hoy hay evidencia concreta de (que el impacto directo e indirecto de la pandemia es mayor en las poblaciones que viven en condiciones de hacinamiento, las minorías étnicas *y* los estratos sociales más bajos, lo cual refleja la desigualdad de la región ^4^.

Ante la magnitud de dicho impacto, la mayoría de los países de la región ha hecho grandes esfuerzos para limitarlo, no solo implementado las medidas de distanciamiento social, sino fortaleciendo la capacidad de los sistemas de salud, la vigilancia epidemiológica y los laboratorios, y suministrando los insumos necesarios con una prontitud sin precedentes en tan corto tiempo ^5^. De todas maneras, la pandemia se encuentra aún en tos estados iniciales en gran parte de la región y los retos que la investigación debe formularse en los siguientes m eses implican la optimización *y* .a focalización de las acciones. En este sentido, el futuro impone la necesidad de promover la investigación científica como base de la respuesta a la pandemia y puente hacia la recup_0_ración social *y* económica de la región ^6^.

En ese contexto, presento aquí tres aspectos fundamentales de la relación entre la investigación científica y la toma de decisiones en la pandemia: el tipo de inversión, las preguntas claves y las áreas prioritarias para su fortalecimiento.

## Inversión en la investigación independiente en contraste con la investigación comisionada

Hay por lo menos, dos ángulos que deben diferenciarse al considerar el tipo de investigación que podría promoverse en Latinoamérica, ambos necesarios para enfrentar la pandemia: en primer lugar, la inversión en la investigación independiente, que implica ofrecer iguales oportunidades a los grupos de investigación para proponer ideas bajo unos lineamientos muy/ generales pero no prescriptivos. Este esquema abre oportunidades tanto para los grupos de trayectoria como para los emergentes y, además, permite la libertad e independencia necesarias para la innovación, lo que es fundamental a la hora de explorar nuevas aproximaciones y encontrar soluciones. En segundo lugar, la inversión en la investigación dirigida o comisionada significa promover los estudios encaminados a resolver problemas o preguntas muy específicas. En el marco de ciertos esquemas, algunos países incluso convocan directamente a los grupos de investigación con trayectoria, infraestructura y capacidad demostradas a producir resultados lo más rápidamente posible.

## Preguntas claves de investigación para orientar las medidas de prevención y control

Sin duda, hay un número importante de preguntas de investigación sobre el SARS-CoV-2 en muchos ámbitos de la ciencia básica ^7^^,^^8^ y en el campo de la salud pública, muchas de las cuales han sido abordadas en la "carta de navegación" para la investigación propuesta por la Organización Mundial de la Salud (OMS) ^9^. Algunas tienen una mayor relevancia que otras en cuanto al tipo de situaciones que determinarán la respuesta frente a la pandemia en los próximos meses en Latinoamérica. Esto porque nuestras condiciones sociales, medioambientales y de exposición a agentes patógenos e, incluso, nuestras características genéticas, difieren de las propias de Asia, Europa o los Estados Unidos. En el cuadro 1 se resumen las que considero de mayor relevancia para la región.

**Cuadro 1:** Preguntas de investigación claves para orientar la respuesta a la pandemia de COVID-19 en Latinoamérica

Pregunta	Diseño
Impacto de la pandemia en la salud pública en Latinoamérica	
¿A qué velocidad y cómo se propaga el virus en espacio y tiempo?	• Análisis de datos de vigilancia rutinaria
	• Estudios de vigilancia activa en diferentes ambientes: trabajo, escuela, ocio, transporte, etc.
¿Cuál ha sido el impacto de las medidas no farmacéuticas en poblaciones y comunidades específicas, incluidas las minorías étnicas?	• Estudios poblacionales probabilísticos de seroprevalencia
¿Cuál es la letalidad del SARS-CoV-2 en Latinoamérica?	• Análisis de registros de mortalidad ligado a estudios de seroprevalencia
¿Cuáles son y qué magnitud alcanzan los efectos indirectos de la pandemia?	• Encuestas poblacionales
	• Encuestas de servicios de salud
	• Análisis de registros
¿Qué condiciones individuales determinan mayor morbilidad y mortalidad asociadas con el SARS-CoV-2?	• Estudios de casos y controles
	• Estudios de cohorte
	• Biomarcadores de la gravedad de la infección
¿Cuáles son los impactos a largo plazo en la salud de los infectados?	• Estudios de cohorte
	
¿Qué condiciones socioculturales determinan un mayor o menor cumplimiento de las medidas preventivas?	• Encuestas poblacionales
	• Estudios cualitativos
¿Cómo cambian las tasas de contacto en Latinoamérica ras la pandemia (es decir, infraestructura de los sitios de trabajo, estudio, costumbres)?	• Encuestas poblacionales
	• Análisis de políticas
	• Estudios cualitativos
Impacto potencial diferenciado de los medicamentos y las vacunas en Latinoamérica	
¿Cuál es el panorama de respuesta inmunitaria frente al SARS-Cov-2?	• Estudios que combinen seroprevalencia y análisis de respuesta inmunológica celular
¿Cuánto perdura la respuesta inmunitaria y qué tan frecuente es la reinfección?	• Estudios de cohorte con mediciones longitudinales de la respuesta inmunitaria en los mismos individuos
	• Caracterización de casos y genómica de la reinfección
¿Qué variantes del virus circulan?	• Vigilancia molecular y genómica con muestras representativas de la evolución espacial y temporal de la propagación del virus
¿Qué variantes genéticas de la población pueden afectar la efectividad de ciertas vacunas?	• Estudios poblacionales de variantes y marcadores genéticos relevantes
¿Cómo garantizar el acceso y la distribución equitativa, oportuna y eficiente de las potenciales vacunas?	• Modelado matemático
	• Estudios de ciencias sociales
¿Cuál es la eficacia de medicamentos y vacunas?	• Ensayos clínicos controlados aleatorizados

## Áreas prioritarias de fortalecimiento de la relación entre la ciencia y la adopción de decisiones

La capacidad de la investigación científica para ganar suficiente relevancia en estos momentos pasa por el fortalecimiento, por lo menos, de los siguientes seis aspectos.

### Sistemas de información y datos libres

La política de datos libres ha sido una consigna de la pandemia, pero los datos libres individualizados todavía son una excepción en la región ^10^. La mayoría de los países de Latinoamérica aún no cuentan con sistemas de vigilancia en salud pública digitalizados, y dependen de fichas manuales que luego deben ser transcritas. Esto genera retrasos para tener disponible la información en tiempo real, así como problemas de calidad de los datos que permean todo el flujo de información. Aquí hay una oportunidad muy importante para que los países hagan inversiones relevantes en el mejoramiento de sus sistemas de información apoyados en la academia y en la industria tecnológica.

### Capacidad analítica y modelado en salud pública en tiempo real

No basta con que los datos sean de acceso libre, tengan calidad y estén disponibles en grandes cantidades, se requieren también la infraestructura y las capacidades para poder analizarlos en tiempo real. La investigación teórica y las capacidades en la ciencia de datos de la academia y la industria han aumentado en Latinoamérica, pero aún hay una brecha en la aplicación de dichas capacidades en los contextos de epidemias y de salud pública. Mediante programas conjuntos de inversión en educación continua por parte de la academia y la industria deben fortalecerse los grupos de análisis y modelado de datos en las instituciones de salud pública, lo cual es un paso fundamental para respaldar la adopción de decisiones ^11^.

### Producción de insumos y equipos básicos

A pesar del fortalecimiento de algunos países en cuanto al número de laboratorios de diagnóstico del SARS-CoV-2, gran parte de la región no ha logrado expandir sus capacidades, en gran medida por problemas en la disponibilidad de insumos ^5^. En junio de este año la Organización para la Cooperación y el Desarrollo Económicos (OCDE) publicó un informe indicando que Latinoamérica es muy dependiente de las importaciones y que menos del 4 % de los productos, equipos médicos, reactivos e insumos básicos para la atención de la pandemia son producidos en la región ^12^. Así que, a pesar del gran esfuerzo desplegado por los países, ha habido serias restricciones de acceso a insumos de diagnóstico y medicamentos y, en un futuro cercano, pasará lo mismo con las vacunas. El fortalecimiento de las capacidades de producción local de insumos, equipos y productos farmacéuticos requiere de la inversión pública y privada.

### Infraestructura para ensayos clínicos, ensayos comunitarios y validaciones

Un estudio publicado en el 2018 indicaba que, aunque las capacidades de ensayos clínicos en Latinoamérica habían aumentado en los últimos años, dicho aumento había ocurrido principalmente en el campo de las enfermedades crónicas, en tanto que para las infecciosas y desatendidas solo había sido del 1 % del total, lo que evidenciaba una discordancia entre la carga de la enfermedad y las capacidades de investigación en este campo ^13^. El apuntalamiento de las capacidades para desarrollar ensayos clínicos y comunitarios en enfermedades infecciosas pasa por fortalecer la formación en investigación clínica, biobancos y comités científicos y de ética en regiones apartadas, con el fin de propiciar la inclusión de la población pobre, diversa y rural. Esto implica la coordinación con las instituciones reguladoras, de manera que, sin perder el rigor científico, se facilite la rápida adecuación de la infraestructura, los procedimientos y los permisos necesarios para desarrollar dichos estudios.

### Comunicación, ética de la investigación y diálogo de saberes

La medicalización de la respuesta frente a la pandemia puede estrechar la visión sobre la forma de evaluar el impacto tal como lo percibe la población. Cuando la población no entiende lo que está sucediendo, se abre el camino a las malinterpretaciones y la desinformación, lo que conlleva un menor cumplimiento de las medidas preventivas. La participación de las ciencias sociales y del comportamiento en los proyectos de investigación desde su concepción misma es esencial para construir puentes entre la comunidad, los responsables de las decisiones y la ciencia. Es así como se crea el marco ético de los proyectos ^14^ y se garantiza el involucramiento de la población en su formulación, tal como se ha propuesto en el caso del desarrollo de las vacunas por su gran impacto ^15^.

### Enfoque regional y cooperación multilateral

No todos los países de la región tienen las mismas capacidades. El papel de liderazgo de las organizaciones regionales, como la Organización Panamericana de la Salud, debe fortalecerse para incentivar una mayor cooperación entre los países en la producción de insumos, medicamentos y vacunas, así como para compartir infraestructura, metodologías exitosas y lecciones.
